# Energy cost of isolated resistance exercises across low- to high-intensities

**DOI:** 10.1371/journal.pone.0181311

**Published:** 2017-07-24

**Authors:** Victor Machado Reis, Nuno Domingos Garrido, Jeferson Vianna, Ana Catarina Sousa, José Vilaça Alves, Mário Cardoso Marques

**Affiliations:** 1 Research Center in Sports Sciences, Health Sciences & Human Development, CIDESD, Vila Real, Portugal; 2 University of Trás-os-Montes & Alto Douro, Vila Real, Portugal; 3 Federal University at Juiz de Fora, Minas Gerais, Brazil; 4 Polytechnic Institute of Viana do Castelo, Viana do Castelo, Portugal; 5 University of Beira Interior, Department of Sport Sciences, Covilhã, Portugal; Nanyang Technological University, SINGAPORE

## Abstract

This study aimed to estimate the energy cost across various intensities at eight popular resistance exercises: half squat, 45° inclined leg press, leg extension, horizontal bench press, 45° inclined bench press, lat pull down, triceps extension and biceps curl. 58 males (27.5 ± 4.9 years, 1.78 ± 0.06 m height, 78.67 ± 10.7 kg body mass and 11.4 ± 4.1% estimated body fat) were randomly divided into four groups of 14 subjects each. For each group, two exercises were randomly assigned and on different days, they performed four bouts of 5-min constant-intensity for each of the two assigned exercises: 12%, 16%, 20% and 24% 1-RM. Later, the subjects performed exhaustive bouts at 80% 1-RM in the same two exercises. The mean values of VO_2_ at the last 30s of exercise at 12, 16, 20 and 24% 1-RM bouts were plotted against relative intensity (% 1-RM) in a simple linear regression mode. The regressions were then used to predict O_2_ demand for the higher intensity (80% 1-RM). Energy cost rose linearly with exercise intensity in every exercise with the lowest mean values were found in biceps curl and the highest in half squat exercise (p<0.001). Half squat exercise presented significant (p<0.001) higher values of energy cost in all intensities, when compared with the remaining exercises. This study revealed that low-intensity resistance exercise provides energy cost comprised between 3 and 10 kcal∙min^-1^. Energy cost rose past 20 kcal∙min^-1^ at 80% 1-RM in leg exercise. In addition, at 80% 1-RM, it was found that upper body exercises are less anaerobic than lower-body exercises.

## Introduction

Resistance exercise (RE) has been progressively more and more popular and is now included in programs which are designed to address weight loss and to target recommended energy cost values [1]. Before time was often supported by non-empirical data about a possible higher energy cost at RE, as compared with typical aerobic activities such as running, cycling or swimming [2]. Comparisons of the excess-post exercise oxygen consumption in RE, with that involved in typical aerobics, showed higher magnitude in RE [3], which also contributed to this belief.

Estimates of the energy cost at RE usually reflect mean values during a whole exercise session (thereby including exercise and recovery periods), ranging considerable between 3 and 11 kcal∙min^-1^ in adult males [4–7]. This large variability is explained by the amount of possible combinations of different exercises, movement cadences, intensities, number of repetitions and also the type of equipment that is used (i.e. machines vs. free weights) [8]. Past research has also addressed the energy costs involved in programs were RE and aerobics combined in the same session, with mean values of 6–7 kcal.min^-1^ while performing the RE, and of 12–14 kcal·min^-1^ while performing the aerobics [7]. In fact, this is now a popular type of program which seems to match most of the populations attending fitness centres. A common ground among the aforementioned studies is the use of typical high-intensity loads (above 70% 1-RM) performed until exhaustion.

Very few studies have addressed the energy cost during isolated RE performed across various intensities. Robergs et al. [9] produced the first paper based in the accumulated oxygen deficit method for RE, combining aerobic and anaerobic estimates from gas exchanges. They studied solely the squat and the bench press exercises, estimating energy cost ranging between 11 and 18 kcal∙min^-1^ and from 8 to 16 kcal∙min^-1^, respectively (40 to 70% 1-RM). Also, Scott and co-workers presented a series of studies on isolated exercises, in which they combine aerobic estimates from gas exchange with anaerobic estimates from blood lactate (50 to 90% 1-RM). Their calculations ranged between 3 and 16 kcal·min^-1^ at bench press [10–12], between 3 and 7 kcal·min^-1^ at biceps curl; and between 6 and 9 kcal·min^-1^ at leg press [10]. In all the above, aerobic energy release during RE was measured through indirect calorimetry, but the anaerobic estimates vary between studies, with the blood lactate energy equivalent being predominant in the literature [10–12].

Current evidence on rate-based energy cost measurements in isolated RE is still scarce, and especially, at low-intensity loads. Despite other models proved more suitable for intermittent RE [13], more data from the typical rate (per minute) energy cost measurements seem needed to foster subsequent counterpoints and different approaches. Due the growing interest of low-intensity RE (i.e. to address the elderly or some pathologies) it is necessary to accumulate data on the specific energy cost of the most popular exercises and, in the future, to use such data to build technology that enables accurate calorie count during RE.

The aim of the present study was to estimate the energy cost across various low-intensities at eight popular resistance exercises: half squat, 45° inclined leg press, seated leg extension, horizontal bench press, 45° inclined bench press, wide grip front lat pull down, standing triceps extension on high-pulley and seated arm curl in Scott bench with Z bar. This was achieved by combining measurements of oxygen uptake and anaerobic estimates by the accumulated oxygen deficit method. It was hypothesized that energy cost would be higher in lower body exercises and that it would rise linearly with intensity.

## Materials and methods

### Participants

A total of 58 males (27.5 ± 4.9 years, 1.78 ± 0.06 m height, 78.67 ± 10.7 kg body mass and 11.4 ± 4.1% estimated body fat), engaged in RE training for at least one year with three or more training sessions per week, volunteered and included the sample. They were recruited among the population engaged in resistance exercise in four fitness centers. Individuals who used medication which could influence their cardiorespiratory response were not included in the sample. After medical approval, the volunteers received the explanations about the procedures, as well as the risks and discomforts involved in the study and signed the written consent form. All procedures were approved by the Review Board of the University of Trás-os-Montes & Alto Douro and were conducted according to the principles expressed in the Declaration of Helsinki. The volunteers were told to refrain from any resistance exercise during the period of the experiment.

### Experimental design

Two exercises were randomly assigned to each subject, so that each and every one was evaluated in two RE. Hence, a total of 15 subjects performed each exercise. All testing was performed in the afternoon (except for the anthropometric measurements), at a temperature between 20-25C° and 35–45% relative air humidity. Each and every subject was submitted to seven testing sessions, as follows.

On the first day, height, weight and several skin folds (chest, mid-axillary, tricipital, sub scapular, abdominal, supra iliac, and thigh) were measured. A calibrated caliper (Lange, Cambridge Scientific Industries, USA) and a digital medical scale with stadiometer (Seca 763, USA) were used for all measurements. Body density was calculated using the equation proposed by Jackson and Pollock [14] and Siri's equation was used to convert the density in percentage of fat mass. These measurements were performed in the morning. In the afternoon of the same day, the subjects performed the 1-RM test at the two assigned exercises using the protocol described elsewhere [15], which was repeated on the second visit (72 hours later). The highest 1-RM with less than 5% difference was considered as the true 1-RM.

On the third to the sixth visit (with 48-hour intervals), the subjects performed (on each visit) two bouts of 4-min constant-intensity exercise -one bout for each of the two assigned exercises. Exercise order for each individual was random and so was the intensity. At each and every RE four intensities were used: 12%, 16%, 20% and 24% 1-RM, amounting a total of four bouts for each exercise All exercises were performed with trademark standardized machines (*Panatta Sport*, Apiro, Italy).

On the last visit (48 hour later) the subjects performed exhaustive bouts at 80% 1-RM in the same two exercises (in random order and with 1-hour recovery between them).

No warm-up was performed before any of the four low-intensity bouts of exercise. Before the 80% 1-RM bout, 2x 15 repetitions with a 24% 1-RM load were used as warm-up, 20 and 10-min before the experimental bout. The cadence of 15 repetitions per minute (2 s on the eccentric and 2 seconds on the concentric phase) was paced by an electronic metronome sound in the four low-intensity bouts. In the higher-intensity bout, cadence was freely chosen by the participant.

### Measurements

During all exercises, expired gases were measured breath-by-breath continuously by open air circuit analyzer (*COSMED® K4b*^*2*^, Rome, Italy). To guarantee accuracy of the gas analysis and minimize respiratory artifacts the participants were instructed avoid unintentional Valsalva maneuvers and inadequate breathing [16]. The gas analyzer was calibrated following the manufacturer's specifications before each testing [17,18]. The mean values of oxygen uptake (VO_2_) at the last 30 s of exercise with 10 s averaging procedure [19] at 12, 16, 20 and 24% 1-RM bouts were plotted against relative intensity (% 1-RM) in a simple linear regression mode. A minimum duration of 4 min was required for a bout to enter this analysis and only steady-state averaging values were included in the regression (variation less than 2 ml·kg^-1^·min^-1^). In addition, a zero-load VO_2_ (individual resting measurement) was also included in the regression by a non-forced procedure. The regressions were then used to predict O_2_ demand for the higher intensity (80% 1-RM). Anaerobic energy release was calculated by the accumulated oxygen deficit method, as explained elsewhere [20]. For final data presentation in this paper, measured O_2_ was converted into energy units (calorie) by a conversion factor of 1 ml O_2_ = 5 calorie.

### Statistical analysis

The sample dimension analysis was performed using G*Power 3.1 software [21]. Under a framework assuming an estimation error of α = 0.05, power = 80%, having 5 measures (intensities) x 8 exercises, an n of 16 was necessary to reach statistical power of 80.8%. Therefore, 20 subjects were initially assigned to each exercise. After drop-out and discard of poor data, the amount per exercise varied between 14 and 17 valid cases. It was decided to favour the same size for each exercise, thereby having a final n = 14. Repeated measures ANOVA was used to analyze energy cost values (8 exercises x 5 intensities), followed by Bonferroni´s post-hoc, whenever necessary. Normality, homogeneity and Sphericity assumptions were confirmed with Shapiro-Wilk, Levene´s and Mauchly tests, respectively. The partial eta squared (η_p_^2^) was used as effect size and interpreted according to Cohen [22]. Overall data for each exercise are presented as means, standard deviations and 95% confidence interval. Significance was set at 5%.

## Results

**Fig 1:**
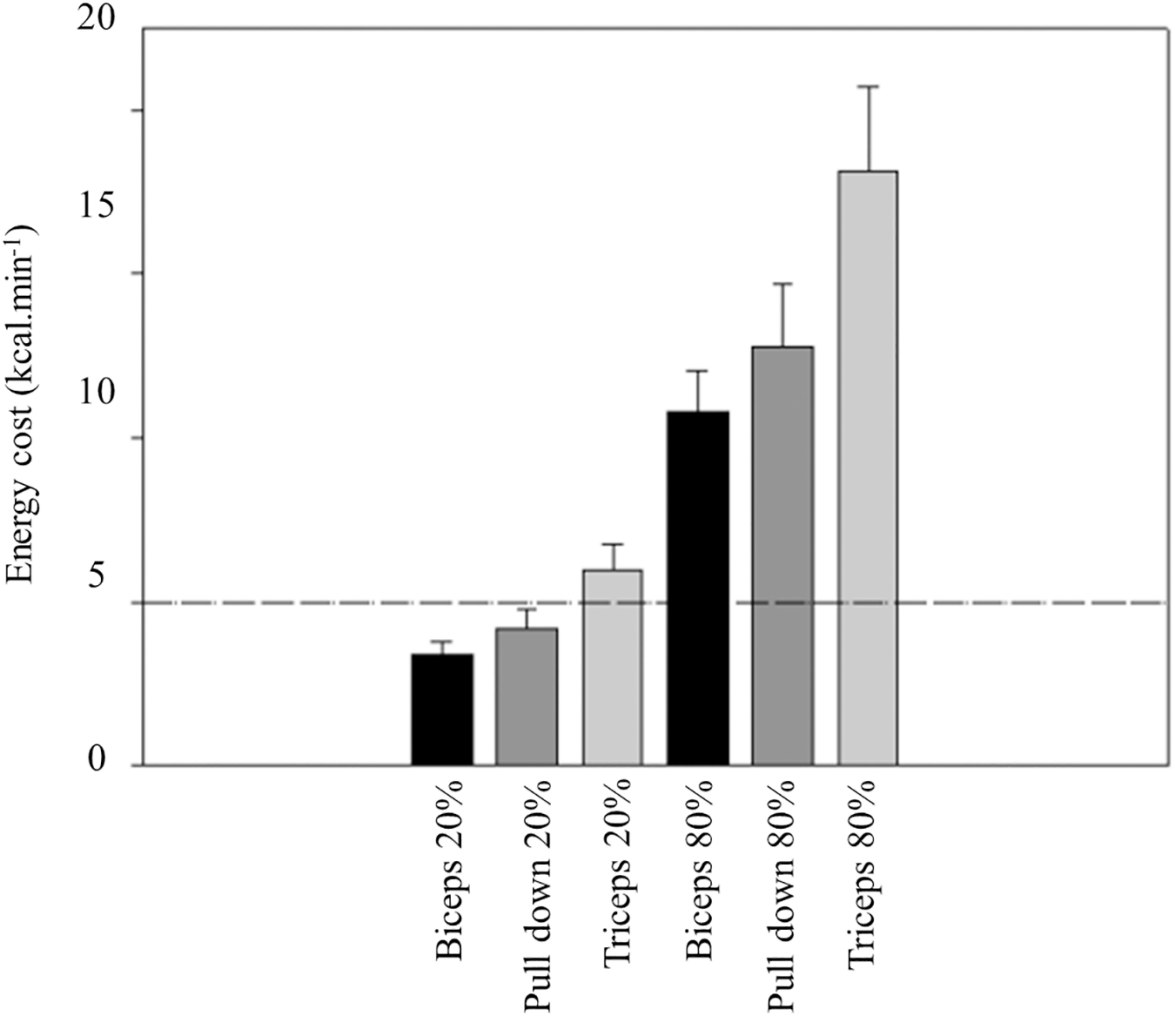
Energy cost (kcal.min^-1^) in two intensities (20% and 80% 1RM) at horizontal and inclined bench press. The 10 kcal·min^-1^ reference line is shown.

**Fig 2:**
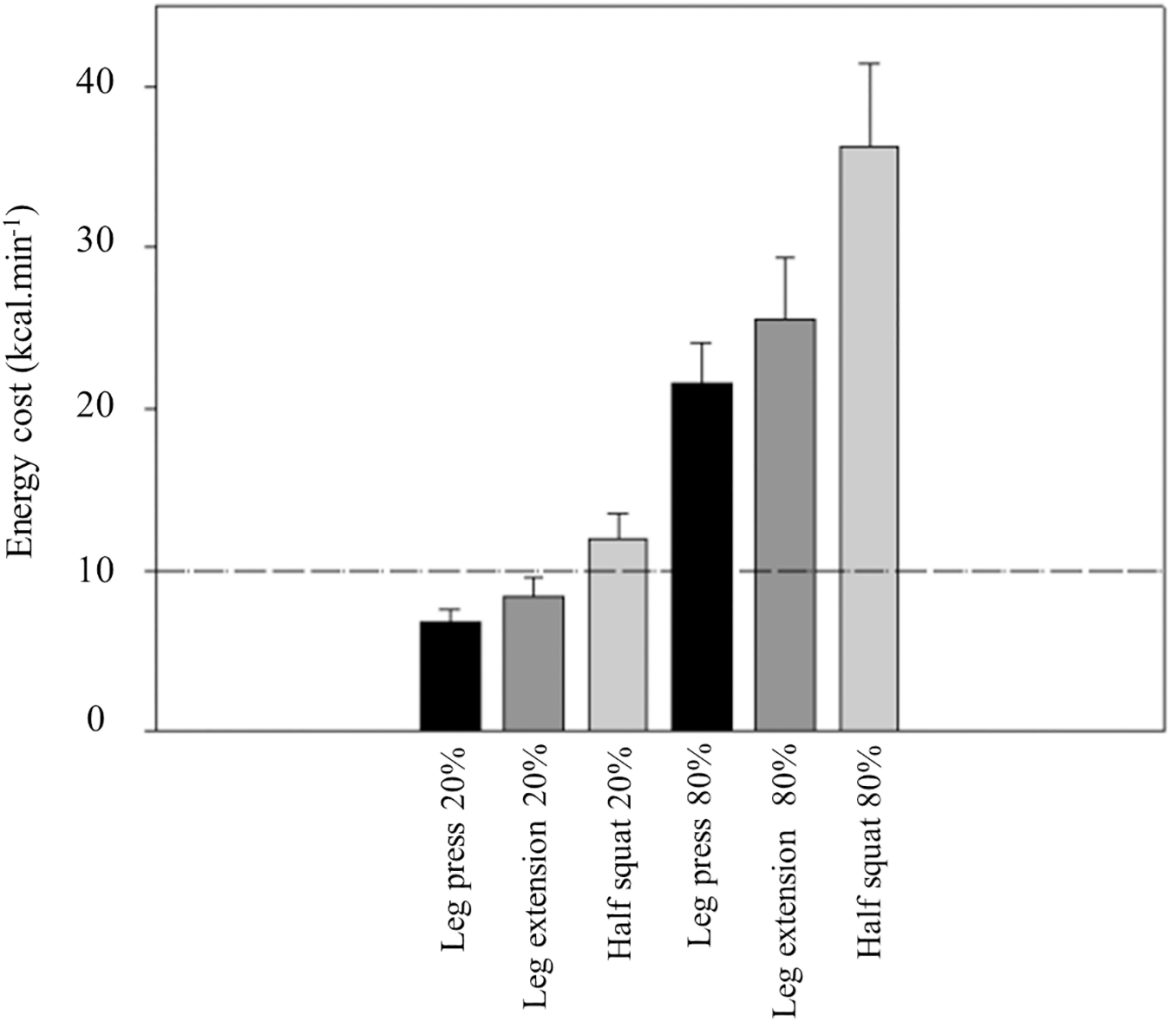
Energy cost (kcal.min^-1^) in two intensities (20% and 80% 1RM) at leg press, leg extension and half squat. The 10 kcal·min^-1^ reference line is shown.

**Fig 3:**
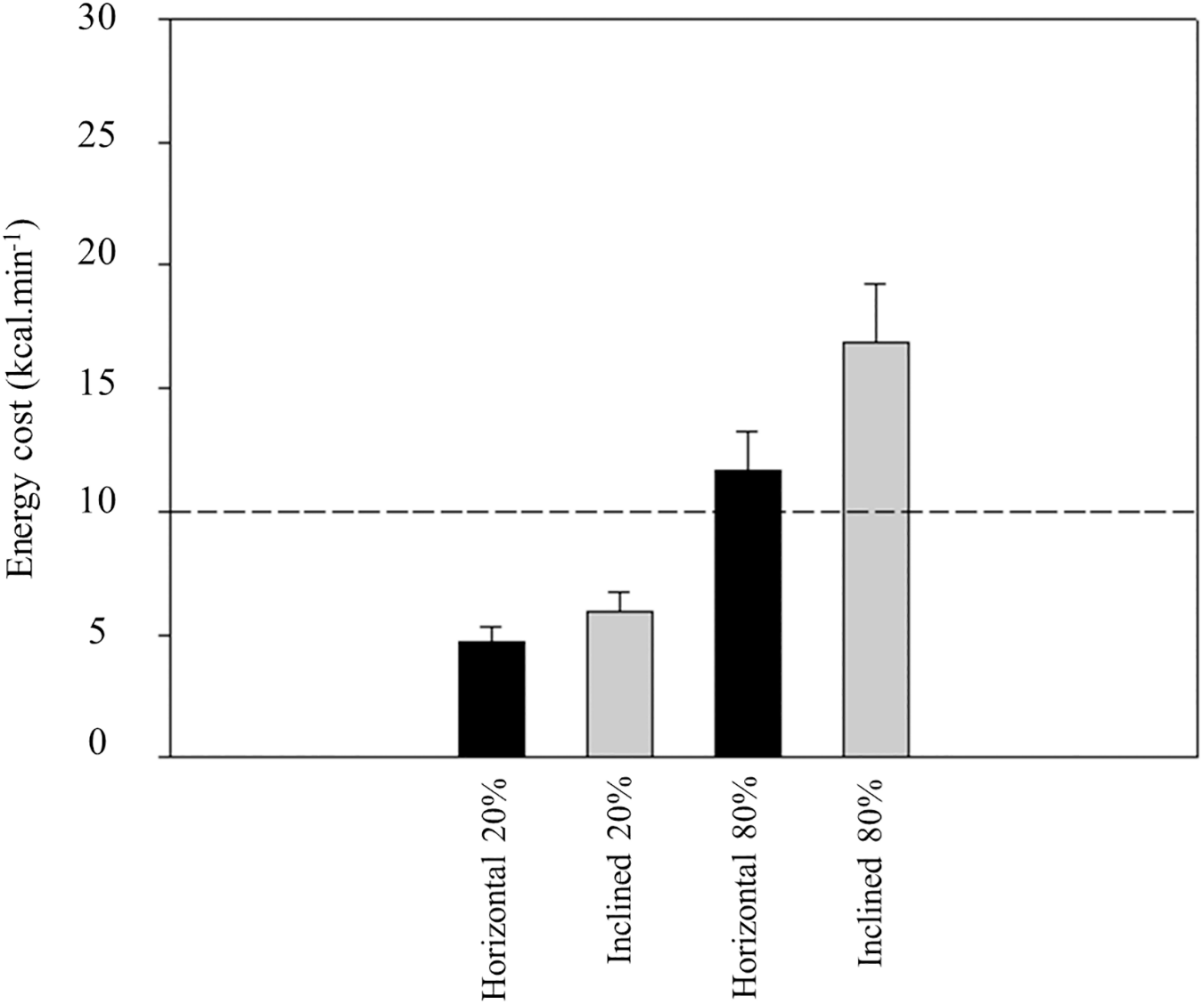
Energy cost (kcal.min^-1^) in two intensities (20% and 80% 1RM) at biceps curl, lat pull down and triceps extension (lower panel). The 10 kcal·min^-1^ reference line is shown.

**Fig 4:**
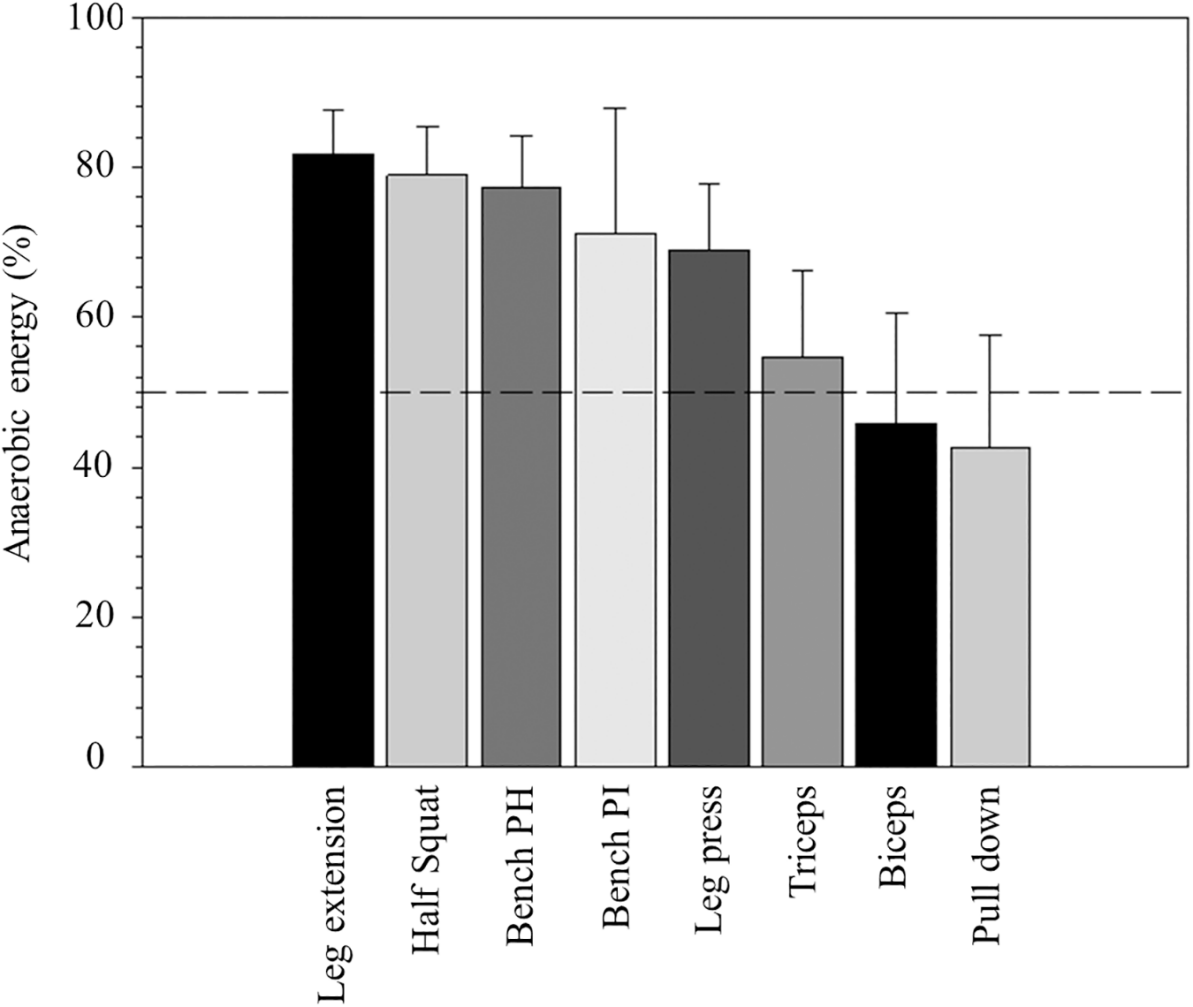
Anaerobic fraction of energy release at the 80% 1-RM bout in the eight different exercises. The 50% reference line is shown. PH = press horizontal; PI = press inclined.

Table 1 presents mean and standard deviations of the energy cost in the eight exercises at the various low intensities. Energy cost increased steadily with exercise intensity in every exercise. The lowest mean values were found in biceps curl and the highest in half squat exercise. It was observed a intensity effect (F_(4, 416)_ = 796.337; p < 0.001; η_p_^2^ = 0.88) and also an exercise effect on energy cost (F_(7, 104)_ = 62.451; p < 0.001; η_p_^2^ = 0.81). A significant interaction exercise x intensity was also found (F_(28, 416)_ = 37.077; p < 0.001; η_p_^2^ = 0.71). Half squat exercise presented significant (p<0.001) higher values of energy cost in all intensities, when compared with the remaining exercises. Table 2 displays data from the high-intensity bout. This was an exhaustive bout and time to exhaustion varied from 26 second in the horizontal bench press and 56 second in the leg press. Energy cost at this intensity presented significant (p<0.001) higher values, when compared with the remaining intensities in every exercise. Fig 1, Fig 2 and Fig 3 depicts energy cost at two intensities (20% and 80% 1RM) in the eight exercises. At the higher intensity, biceps curl was the single exercise with mean values below 10 kcal∙min^-1^, whereas energy cost attained values above 30 kcal∙min^-1^ in half squat (η_p_^2^ = 0.81). Fig 4 shows the anaerobic fraction of energy release at the 80% 1-RM bout in the eight exercises. Aerobic energy was predominant in biceps curl and in front lat pull down.

**Table 1:** Energy cost (kcal·min ^-1^) and absolute load (kg) at low intensities in the eight exercises. Values are mean ± standard deviation (95% confidence interval for energy cost).

	12% 1RM	16%1 RM	20% 1RM	24% 1RM
H Bench press Load	3.99 ± 0.56 (3.65–4.24)	4.07 ± 0.57 (3.72–4.39)	4.67 ± 0.65 (4.10–5.04) #	4.83 ± 0.67 (4.22–5.14) +
	11.35 ± 2.57	15.18 ± 3.82	18.94 ± 4.28	22.65 ± 4.97
I Bench press	5.21 ± 0.75 (4.45–5.56) ω	5.65 ± 0.81 (4.78–6.22) χ	5.91 ± 0.85 (4.80–6.72) β+	6.52 ± 0.94 (5.12–7.17)?
Load	10.21 ± 2.12	13.62 ± 2.82	17.02 ± 3.52	20.43 ± 4.23
Half squat	10.06 ± 1.55 (9.35–11.40) $	10.78 ± 1.51 (9.80–11.39) $	11.70 ± 1.67 (10.66–12.48)$#	11.90 ± 1.64 (10.61–12.42)$?
Load	13.88 ± 3.24	18.82 ± 4.02	23.05 ± 5.01	28.29 ± 6.21
Leg press	5.02 ± 0.60 (5.50–6.59) *f*	5.91 ± 0.71 (5.73–7.35) *f*	6.74 ± 0.81(6.60–8.21) *f*+	7.32 ± 0.88 (7.11–9.56) +
Load	33.60 ± 4.62	48.12 ± 7.43	61.66 ± 9.02	72.73 ± 10.96
Leg extension	6.51 ± 0.94 (5.63–7.04) ϕ	7.82 ± 1.12 (6.70–8.51) ψ#	8.31 ± 1.20 (5.88–8.63) Ω	8.92 ± 1.28 (6.30–10.34) *f*
Load	17.88 ± 2.97	23.83 ± 2.93	28.42 ± 4.35	36.08 ± 6.51
Latt pull down	3.39 ± 0.47 (2.96–3.52)	3.35 ± 0.46 (2.82–3.53)	4.03 ± 0.56 (3.54–4.25)	4.51 ± 0.63 (3.59–5.15)
Load	11.24 ± 1.60	14.82 ± 2.10	18.47 ± 2.83	22.24 ± 3.44
Biceps curl	2.68 ± 0.33 (2.95–3.62)	3.15 ± 0.37 (3.05–3.99)	3.42 ± 0.41 (3.50–4.36) #	3.87 ± 0.46 (3.81–4.85) #
Load	4.27 ± 0.32	6.15 ± 0.93	7.67 ± 1.26	9.29 ± 1.51
Triceps ext	3.27 ± 0.45 (2.73–3.68)	3.47 ± 0.48 (3.05–3.96)	4.31 ± 0.60 (3.58–4.92) #	4.83 ± 0.67 (4.12–5.34) +
Load	5.53 ± 1.12	7.41 ± 1.58	9.41 ± 1.87	11.24 ± 2.39

H = horizontal; I = inclined; ext = extension.

ω—p<0.02 compared with all exercises with exception of H Bench press and Leg press

χ—p = 0.001 compared with Biceps curl

*f*—p<0.0001 compared with all exercises with exception of I Bench press and Leg press

ϕ—p<0.02 compared with all exercises with exception of Leg press

$—p<0.0001 compared with all exercises

ψ—p<0.0001 compared with all exercises with exception of the Leg press

#—p<0.05 compared with 12% 1-RM; β—p<0.03 in relation to Lat pull down, Biceps curl and Triceps ext

+—p<0.05 compared with 12% and with 16% 1-RM

Ω—p<0.0001 compared with all exercises with exception of I Bench press and Leg press

?–p<0.01 compared with 20% 1-RM

&—p<0.0001 compared with all exercises with exception of I Bench press.

**Table 2:** Energy cost, absolute load (kg), repetitions, time to exhaustion and total volume (load x repetitions) at 80% 1-RM in the eight exercises. Values are mean ± standard deviation (95% confidence interval for energy cost).

	Energy cost (kcal·min ^-1^)	Load (kg)	Repetitions	Time (min)	Volume (kg)
H Bench press	11.41 ± 2.35 (10.05–12.76) *	75.18 ± 16.37	8.64 ± 2.25	0.44 ± 0.10	649.6 ± 36.8
I Bench press	16.77 ± 6.06 (13.28–20.27) χ *	68.08 ± 15.02	11.00 ± 3.12	0.73 ± 0.21	748.9 ± 46.9
Half squat	35.94 ± 4.98 (33.07–38.81) $ *	94,59 ± 20.43	11.55 ± 3.59	0.47 ± 0.20	1092.5 ± 73.3
Leg press	19.86 ± 4.83 (17.07–22.65) € *	242.69 ± 39.84	14.11 ± 2.69	0.94 ± 0.18	3424.4 ± 107.2
Leg extension	25.70 ± 9.23 (20.37–31.04) Υ*	119,17 ± 19.79	8.00 ± 1.40	0.55 ± 0.08	953.4 ± 27.71
Latt pull down	9.58 ± 2.76 (7.98–11.17) *	74.24 ± 10.87	11.09 ± 1.87	0.50 ± 0.06	823.3 ± 20.3
Biceps curl	8.53 ± 2.25 (7.23–9.83) *	30.76 ± 4.97	12.70 ± 3.75	0.85 ± 0.25	390.7 ± 18.6
Triceps extension	10.86 ± 3.29 (8.96–12.76) *	37.07 ± 7.77	11.55 ± 4.27	0.48 ± 0.15	428.2 ± 33.2

H = horizontal; I = inclined.

*—p<0.001 compared with all lower intensities

χ—p = 0.001 compared with Biceps curl

$—p<0.001 compared with all exercises

€ - p<0.001 compared with all exercises with exception of I Bench press and Leg extension

Υ - p<0.03 compared with all exercises with exception of Leg press.

## Discussion

The aim of the present study was to estimate the energy cost in resistance exercises performed at low intensities (12%, 16%, 20%, 24% 1RM) during 4-min steady-state exercise; Moreover, the former estimates were extrapolated for 80% 1RM exercise intensity. From the eight popular resistance exercises selected for this study, the half squat and leg extension were the ones which involved higher energy cost (~11 and ~8 kcal·min^-1^, respectively), contrasting with the biceps curl and lat pull down (~3 and ~4 kcal·min^-1^, respectively) at all intensities studied. In addition, we can conclude that, even at a high intensity– 80% of 1-RM, energy cost of the upper limbs may be mainly aerobic whereas those of the lower limbs are evidently anaerobic.

### Energy cost at lower intensities

The majority of studies conducted used a typical high-intensity loads (above 70% 1-RM), and therefore, very few studies have addressed the energy cost during isolated RE performed across various low-intensities. To the best of our knowledge, a single study [9] used a similar method to this study (linear extrapolation of RE using 4 or 5-minute steady state values of oxygen uptake) reporting a energy cost of 8 kcal·min^-1^ for bench press and 11 kcal·min^-1^ for squat at 40% 1RM. In the present study, we found lower values at intensities up to 24% 1RM: 4.8 kcal·min^-1^ for horizontal bench press and 6.5 kcal·min^-1^ for inclined bench press. However, the value reported for squat exercise (11.7 kcal·min^-1^) corroborated the previous suggested. Buitrago et al. [23], though with chest press machine exercise, provided evidence in favour of the linearity of power and energy cost, especially with aerobic energy cost. Although using a different method—peak blood lactate accumulation post effort, some reported similar energy cost values to ours: 2.7, 5.3 and 7.26 kcal·min^-1^when performing bench press at 50% 1RM with 7, 14 and 21 repetitions, respectively [11]. In the present study, the eight RE showed values between 3.87 kcal·min-^1^ and 11.70 kcal·min^-1^ when performed at low intensity (24% 1-RM). A circuit RE including exercises at moderate intensity (~43% 1-RM) also showed energy cost ~9 kcal·min^-1^.[24] Collectively, these studies suggest that even at low intensities, RE could be an efficient method to weight loss purposes. In fact, typical reference values of 3 METs and 8.5 METs are described for walking at 4km·h^-1^ and running at 8 km·h^-1^, respectively, in one subject with body weight similar to those in the present study [25].

### Energy cost and anaerobic energy release at high-intensity

When using O_2_ demand to predict energy cost at the higher intensity (80% 1-RM) by simple linear regression mode, we found values that are close to those reported with the same method [9] in bench press exercise at 70% of 1-RM. However, when the squat exercise is taking into account, our values are 50% higher than those reported (36 kcal·min^-1^ vs.18 kcal·min^-1^, respectively). The large variation of the results found could be related, in one hand, with of the standard error of the regression line between VO_2_ and work. In fact, this latter was larger in the half squat (~19 ml·kg·min^-1^), compared with the other exercises. On the other hand, the different intensities used in the studies (70% and 80% of 1-RM) and difference between subjects- although we watched carefully for variations in technique or rate, each subject can present different changes with volume, intensity or fatigue, may have contributed to the differences found between studies. Although not yet investigated in RE, the lack of linearity between work rate and oxygen uptake throughout all intensities, due to some potential mechanics (intensification of respiratory muscle activity increased muscle temperature, increased activation of additional muscle groups, recruitment of type II muscle fibers, lactate and proton accumulation) may have also played an important role [26].

The accumulated oxygen deficit allows the quantification of the aerobic and anaerobic fraction of energy release in relation to the overall energy cost. This method, rarely used in RE, is considered as the most reliable available measure of anaerobic energy release during high intensity exercise [27]. In RE the quantification of anaerobic energy release by the blood lactate equivalent has been more popular [10–12]. However, blood lactate on and off-kinetics during RE is still poorly understood, and therefore prone to the several sources of error pointed out for cycling [31]. Moreover, this method also requires another assumption (not measure) as to the alactic fraction of energy release.

In the present study, the anaerobic fraction of energy release at the 80% of 1-RM presented a higher percentage in the majority of the exercises, as expected, due to limited ATP and creatine phosphate stores within working skeletal muscle [13]. However, the biceps curl and lat pull down exercises had an anaerobic fraction under 50%, with the triceps extension exercise showing a little up of the reference line. Collectively, these results suggest that even at a higher intensity, energy cost of the upper limbs may be mainly aerobic whereas those of the lower limbs are evidently anaerobic. Considering the latter, we suggest that a larger recovery is needed after lower limb exercises for similar exercise intensity.

Not consistent with this hypothesis, is the horizontal and 45° inclined bench press exercises. In fact, the anaerobic energy cost of the latter was ~13 and 17 kcal·min^-1^ (respectively) representing from 70 to 77% of total energy release, which confirms previous data [12] at 37 to 90% 1-RM. This apparent inconsistency could be related with the muscle mass involved. Therefore, when upper limbs exercises involve small muscle masses, a higher fraction of aerobic energy seems to be present, and vice versa. This fact suggests that not only which members are involved in the exercise, attention must be driven also to the amount of muscle mass involved. Moreover, the upper-body has a higher proportion of fast-twitch fibres [28], being these related with an increased inefficiency compared with lower body-exercise [29]. In fact, Muraki et al. [30] by studying *triceps brachii* muscle oxygen saturation using Near Infra Red Spectometry during arm cranking and cycling exercise in young women, noted a faster increase in the respiratory exchange ratio and a lower ventilatory threshold in arm compared with leg exercise, suggesting accelerated anaerobic glycolysis. Notwithstanding the previously mentioned, the potential mechanism behind the energy cost in RE involving both lower and upper body limbs, with small and higher fractions of total muscle mass has, to date, not been investigated.

This study revealed that resistance exercise may provide energy cost compatible with weight loss purposes, even if low-intensity and large-volume workout is performed. In addition, at high-intensity, is seems that upper body exercises are less anaerobic than lower-body exercises. This better knowledge about energy cost during RE can help professionals to predict accumulated energy cost during a session based in the eight popular exercises herein. In the future and with further data on various exercises and various populations, hopefully one can accurately design new technology (wearable or attached to weight machines) that enables accurate calorie count during resistance exercise.

Despite the fact that the accumulated oxygen deficit method does not requires measurements of low-intensity blood lactate, this may be viewed as a possible limitation of this experiment. The analysis herein can be improved, at least theoretically, by including low-intensity blood lactate measures. The results herein were obtained with a population of male, apparently healthy well-trained individuals and therefore may not apply to less-trained individuals or to special other populations.

## Conclusions

The results herein confirm the hypothesis that energy cost during resistance exercise rises linearly with intensity. They also confirm the hypothesis that lower body exercise present higher energy cost when compared with upper body exercise. Half squat and leg extension involved the highest energy cost (~11 and ~8 kcal·min^-1^, respectively), contrasting with biceps curl and lat pull down (~3 and ~4 kcal·min^-1^, respectively). These values refer to low-intensity exercise (between 12 and 24% 1-RM).

## Supporting information

S1 Dataset Dataset used in this research. (XLSX)
